# A Review of Nuclear-Organellar Communication in Response to Heat Stress

**DOI:** 10.3390/plants15192995

**Published:** 2026-09-30

**Authors:** Linlin Zhang, Xiaofei Li, Ankun Pan, Xueming Su, Long Chen, Tianxiao Chen, Baohua Feng, Shen Ni

**Affiliations:** State Key Laboratory of Rice Biology and Breeding, China National Rice Research Institute, Hangzhou 310006, China

**Keywords:** heat stress, nuclear-organellar communication, plasma membrane, nucleus, chloroplasts, mitochondria, endoplasmic reticulum

## Abstract

Plants coordinate their growth and developmental programs through dynamic communication in the core of the nucleus, enabling them to adapt to persistently high-temperature environments. Upon heat stress, plants primarily perceive and transduce thermal signals between the nucleus and other organelles, a process defined as nuclear-organellar communication. The published research on plants in response to heat environments mainly focuses on sensing heat physical signals from the plasma membrane (PM)/cytosol to the nucleus, eliciting nuclear transcriptional reprogramming and decoding organelle-derived retrograde signals to the nucleus. Consequently, plants have evolved sophisticated systems to decode adverse temperature signals relying on efficient nuclear-organellar coordination. This review summarizes how plants maintain cellular homeostasis via nuclear crosstalk. Heat signals are initially perceived, which triggers the production of secondary messengers including phosphatidic acid (PA), calcium ions (Ca^2+^) and reactive oxygen species (ROS). In addition, proteins also deliver thermal signals to the nucleus. As the central signaling hub, the nucleus orchestrates global transcriptional reprogramming predominantly through the Heat Shock Transcriptional Factors (HSFs)–heat shock proteins (HSPs) regulatory module, and sends instructions to the endoplasmic reticulum (ER), chloroplasts and mitochondria. Conversely, organelle-derived retrograde signals coordinate stress adaptation: the unfolded protein response relieves ER stress, and heat stress activates ROS-mediated chloroplast-to-nucleus and mitochondria-to-nucleus feedback pathways. We further outline candidate thermosensing proteins residing in different organelles, and highlight major unsolved issues. Critical knowledge gaps include the intrinsic thermosensing capacity of chloroplasts and mitochondria, inter-organellar coordination of second-messenger dynamics, specific organelle functions, and the biological roles of membrane contact sites. Further mechanistic insights will facilitate the application of genome editing, synthetic biology and artificial-intelligence-aided design to breed heat-tolerant crops without yield penalties.

## 1. Heat Signal Perception and Transmission to the Nucleus

**Figure 1:**
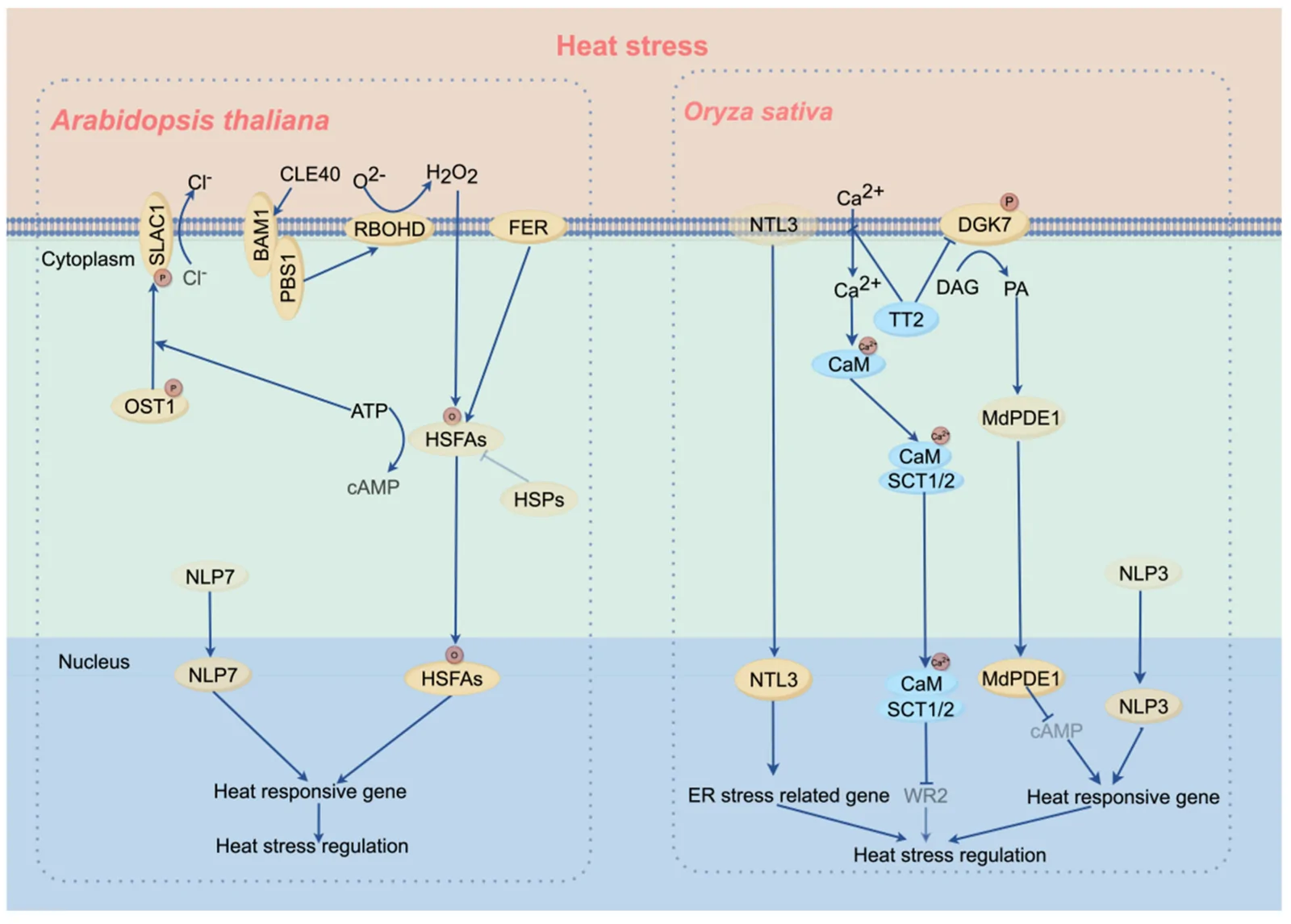
Heat signals are sensed and transferred through chemical molecules and proteins. In *Arabidopsis thaliana*, heat triggers oxidative stress and activates HSFAs to translocate into the nucleus. This process facilitates the expression of heat-responsive genes and removes the repression of OST1, thereby promoting OST1-dependent stomatal closure. Meanwhile, activated NLP7 also relocates to the nucleus, upregulating heat-responsive genes to improve thermotolerance. In *Oryza sativa*, heat stress triggers Ca^2+^ accumulation, which represses *WR2* expression via CaM-SCT1/2, thus negatively regulating heat stress. Heat also stimulates PA production mediated by PM-localized DGK7 to further induce heat-responsive gene expression. PM-localized protein NTL3 translocates to the nucleus to modulate the expression of ER stress-related genes, ultimately improving plant thermotolerance. This figure was made by Figdraw 2.0 software.

Thermosensing represents the initial step in the heat stress response [1,2,3]. Under heat stress, modifications of plasma membrane (PM)-localized proteins facilitate the accumulation of secondary messengers in the cytosol, including phosphatidic acid (PA), calcium ions (Ca^2+^), and hydrogen peroxide (H_2_O_2_). Notably, the increase in these chemical molecules triggers responses via cascade signal transduction. Furthermore, proteins that alter their subcellular localization or undergo conformational changes can also function as thermosensors to maintain homeostasis. This process allows adaptation to adverse high temperatures (Figure 1). As a result, plants respond to heat waves at multiple levels to ensure survival. However, the complex regulatory networks between the plasma membrane (PM)/cytosol and the nucleus remain poorly understood. In this review, we summarize how plants sense heat signals, particularly focusing on their interplay with the nucleus.

### 1.1. Chemical Molecules at the PM Transducing Heat Signals

The PM is highly sensitive to temperature fluctuations. Heat stress induces hyperfluidization of PM, which disrupts membrane integrity and accelerates ion leakage, ultimately leading to cell death. Therefore, stabilizing PM fluidity is a critical determinant of plant thermotolerance [4]. Furthermore, heat-induced modification or conformational changes in PM-localized proteins via chemical molecules also play essential roles in nuclear-organellar signal transmission.

In rice, a cascade regulatory pathway from sensing at the PM to transcriptional reprogramming in the nucleus has been revealed to decode heat signals. Heat activates PM-localized protein Diacylglycerol Kinase 7 (DGK7) to convert diacylglycerol into PA in the cytosol. Subsequently, Metal-Dependent Phosphodiesterase 1 (MdPDE1) directly binds PA and translocates into the nucleus, where it degrades cyclic adenosine monophosphate (cAMP), thereby reducing cAMP and triggering transcriptional reprogramming that promotes the gene expression of small heat shock proteins (sHSPs) and reactive oxygen species (ROS)-scavenging enzymes adapted for heat stress. Moreover, this pathway is negatively regulated by the G protein subunit TT2 (Thermotolerance 2), which dephosphorylates DGK7 at Ser477 to reduce function [5]. Collectively, DGK7 is a potential thermosensor; several questions remain to be investigated. It is unclear whether heat directly induces DGK7 autophosphorylation through structural changes or via intermediate factors. Furthermore, the mechanism by which TT2 mediates DGK7 dephosphorylation requires further investigation.

In addition to PA-mediated regulation, Ca^2+^ serves as a universal second messenger for rapid signal amplification. Heat stress induces rapid Ca^2+^ influx across the PM, leading to cytosolic Ca^2+^ signatures that trigger downstream biochemical responses, including the expression of *heat shock proteins* (*HSPs*) [6,7]. A Recent finding indicates that a natural allele with loss of Gγ protein Thermotolerance 2 (TT2) function represses Ca^2+^ influx under heat conditions and enhances thermotolerance in rice. This process weakens the interaction between calmodulin (CaM)-Sensing Ca^2+^ Transcription Factor 1/2 (SCT1), which relieves the inhibitory effect on Wax Synthesis Regulatory 2 (OsWR2), thereby positively regulating the heat stress response [8]. Although establishing a bridge linking G protein, Ca^2+^ sensing and wax metabolism, in contrast to mammals, where G proteins directly bind to ion channels [9], this direct interaction has not been identified in plants.

Besides PA and Ca^2+^, ROS also function as early signals, characterized by an ultra-rapid ROS burst upon heat stress. However, the upstream mechanism by which plants sense high temperature and subsequently trigger ROS production remains poorly elucidated in previous studies. Currently, emerging evidence indicates that in Arabidopsis, the receptor-like kinases BARELY ANY MERISTEM 1 (BAM1) and AVRPPHB SUSCEPTIBLE 1 (PBS1) form a complex that activates the NADPH oxidase Respiratory Burst Oxidase Homolog Protein D (RBOHD) upon heat stimulation, triggering H_2_O_2_ production in the cytosol. H_2_O_2_ acts as a second messenger, in which the master Heat Shock Transcription Factor A1b (HSFA1b) undergoes oxidative modification and subsequently translocates into the nucleus to induce the expression of heat-responsive genes. The secreted peptide CLAVATA3/EMBRYO SURROUNDING REGION-related 40 (CLE40) reinforces BAM1–PBS1 complex formation, creating a positive feedback loop to sustain heat signal transduction [10,11]. This finding highlights that BAM1-PBS1 at the PM may function as an early-acting component in plant heat signaling. However, the mechanism of heat-triggered BAM1 activity remains poorly understood.

In summary, heat triggers the accumulation of chemical molecules in the cytosol, which subsequently regulate signaling to the nucleus. Recent studies indicate that PM-localized proteins convert thermal signals into chemical messengers (PA, Ca^2+^, and ROS), which are then relayed via protein cascades to elicit transcriptional reprogramming in the nucleus. Although these signal transduction pathways have been reported, the direct mechanisms activating these proteins remain largely unknown. Future studies should examine whether proteins undergo heat-induced conformational changes or directly interact with ion channels to enhance their activity. In addition, the overaccumulated chemical molecules are detrimental to plant cells; further research requires the braking mechanisms to sustain homeostasis.

### 1.2. Proteins Delivering Messages to the Nucleus

Beyond soluble signals, dual-localized proteins directly mediate communication with two or more organelles by shuttling between compartments. Accumulating evidence suggests that PM-localized proteins receive heat signals and subsequently relocalize to exert functions in the nucleus. For example, the PM-associated transcription factor NAC domain transcription factor 3 (OsNTL3) may act as a potential sensor during heat stress. It relocates to the nucleus to elevate the expression of *OsbZIP74* and maintain ER homeostasis in response to heat stress [12]. Currently, it remains elusive whether heat-induced translocation relies on intrinsic conformational changes or proteolytic cleavage.

Several cytosolic proteins have been identified as mediators of nuclear-organellar communication under heat stress. For instance, the nuclear localization of Heat Shock Transcriptional Factors (HSFs) is critical for plant responses to heat stress. Under normal temperatures, HSFs bind to heat shock proteins (HSPs) including HSP70 and HSP90 to form an inactive complex in the cytosol. Upon heat shock, HSPs bind to misfolded/unfolded proteins, thereby releasing HSFs that translocate into the nucleus to boost plant thermotolerance. The dual subcellular localization of HSFs suggests that disrupted protein homeostasis acts as a rapid signal relayed to the nucleus [13]. This evidence implies that heat perception is initiated by damaged ER-resident proteins, which may function as potential heat sensors. In addition, the cytoplasmic nitrate sensor, a NIN-like protein (NLP3), translocates to the nucleus in response to heat stress, particularly under nitrate-replete conditions, where it modulates the expression of heat-responsive genes to maintain seedling survival [14]. Consistent findings in Arabidopsis demonstrate that NLP7 senses heat signals and moves into the nucleus to promote *HSFA3* expression and improve thermotolerance [15]. Collectively, these findings indicate that nitrate signaling enhances heat resistance through a conserved mechanism in both rice and Arabidopsis. However, how NLP3 or NLP7 rapidly responds to heat signals is poorly understood, and identifying additional regulatory factors acting upstream of NLPs remains an important research objective. Intriguingly, in the cytosol, HSFA1b inhibits Open Stomata 1 (OST1) kinase activity through its intrinsic adenylate cyclase activity, which catalyzes cAMP biosynthesis. The accumulated cAMP in turn restrains stomatal closure by suppressing OST1 activity. Under heat stress, HSFA1b translocates into the nucleus, relieving OST1 inhibition. De-repressed OST1 subsequently triggers Slow Anion Channel 1 (SLAC1) activation to drive stomatal closure. Simultaneously HSFA1b activates *HSP* expression to enhance heat tolerance [16]. Collectively, dual-localized proteins exert vital regulatory functions in plant response to heat stress. Nevertheless, the upstream molecular regulators governing the localization and activity of these proteins remain largely uncharacterized and require deeper experimental investigation.

In Arabidopsis, recent articles have uncovered a mechanism analogous to that of animal TRP channels. The malectin-like receptor kinase FERONIA (FER) acts as a membrane-anchored thermal switch. Moderate heat activates FER through the RAPID ALKALINIZATION FACTOR 34 (RALF34) peptide and promotes its recruitment to sterol-rich nanodomains. These nanoclusters stabilized liquid-ordered membrane phases and activated the Heat Shock Response (HSR) signaling in the nucleus, enhancing thermotolerance [17].

Emerging evidence identifies numerous proteins that traffic between organelles under both normal and heat stress conditions. However, whether these proteins directly perceive heat signals or merely relay downstream messages remains largely unknown. Future research must delineate these upstream signaling events to pinpoint the primary thermosensors.

## 2. The Central Hub Nucleus Cascading Heat Signals to Other Organelles via Transcriptional Regulation

**Figure 2:**
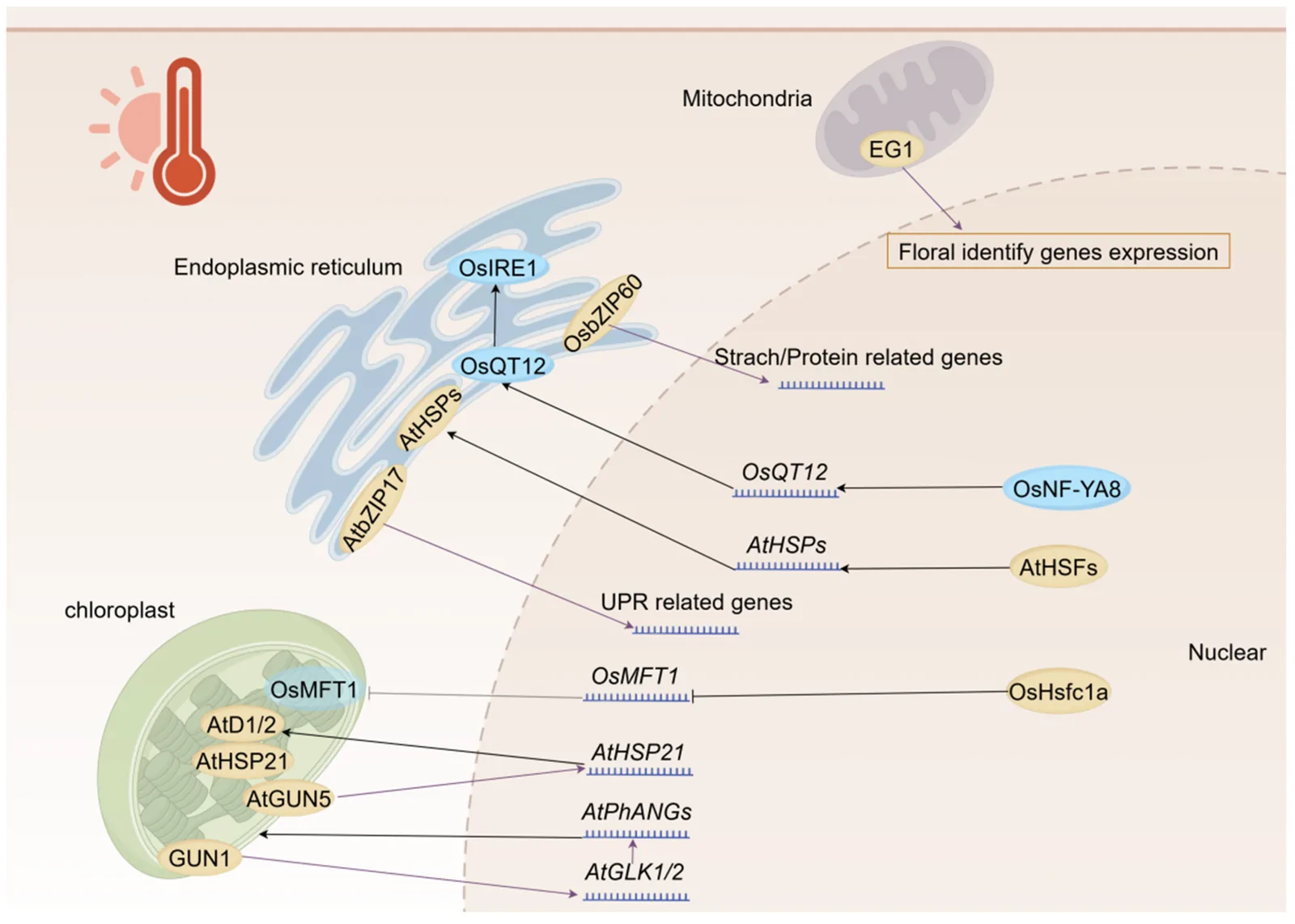
The transduction of organelles during heat stress. Inside the chloroplast, protective mechanisms are activated to maintain organelle function. OsHsfc1a represses *OsMFT1* to prevent its excessive accumulation, which could otherwise damage chloroplast integrity. Meanwhile AtGUN1/5 mediate the communication between the chloroplast and nucleus. Additionally, AtRPS1 localized in chloroplast promotes the expression of AtHSFA2. In the endoplasmic reticulum (ER), the heat signal allows OsNF-YA8 to upregulate *OsQT12* expression, thereby modulating the unfolded protein response (UPR) and damage to the ER. Concurrently, OsbZIP60 and AtbZIP17 translocate from the ER to the nucleus, where they upregulate gene expression, promoting cellular adaptation to heat. Within mitochondria, EG1 enhances the expression of floral identity genes, contributing to developmental adjustments under thermal stress. This figure was made by Figdraw 2.0 software.

The nucleus serves as the central hub for integrating heat signals and mediating global changes in gene reprogramming. The heat stress process, predominantly controlled by HSFs and HSPs, is highly conserved and essential for enhancing thermotolerance [18,19]. Upon heat stress, organelles relay thermal signals to the nucleus, where HSFs are activated and promote the expression of heat-responsive genes that, in turn, regulate the function of other organelles (Figure 2). This rapid signal transmission is critical for plant survival. Therefore, understanding how the nucleus transmits instructions to other organelles is pivotal for deciphering thermotolerance mechanisms. This section summarizes the communication mainly from the nucleus to other organelles via transcriptional cascade regulation.

Recent advances report that HSFs also function in other organelles via transcriptional regulation. For example, OsHsfc1a is strongly induced by heat stress and represses *MOTHER OF FT AND TFL1* (*OsMFT1*) expression, thus protecting chloroplast structural integrity against excessive OsMFT1 protein accumulation. The OsHsfc1a-OsMFT1 module establishes a nucleus-chloroplast regulatory circuit critical for heat adaptation [20]. OsMFT1 encodes a phosphatidylethanolamine-binding protein; however, its function remains poorly understood. In addition, Heat-induced expression of the nuclear-encoded HSP21 leads to the import of HSP21 protein into chloroplasts, where it protects Photosystem II components D1 and D2 via a Genomes Uncoupled 5 (GUN5)-dependent mechanism [21]. Substantial progress has recently been made in deciphering the regulatory mechanisms of HSFs and HSPs. This suggests that nuclear-organellar signaling plays an essential role in plant responses to heat stress. Still, the unique transcriptional transduction cascades await in-depth exploration. Likewise, post-translational modifications including oxidation and acetylation merit systematic investigation. Additionally, the exact biological functions of organelle-localized HSPs remain poorly characterized.

Beyond the core HSFs regulatory pathway, recent articles also find other factors that maintain organelle homeostasis. For example, high temperatures weaken the interaction of NF-YB9/NF-YC10 with NF-YA8. This process releases *Grain Quality-Thermotolerant QTL on Chromosome 12* (*QT12*) suppression; thus, QT12 disrupts endosperm storage substance homeostasis by over-activating UPR, triggering quality deterioration [22]. This model establishes NFs complex regulates protein homeostasis in the ER via QT12 transcriptional regulation. However, the mechanism by which QT12 mediates IRE1 phosphorylation deserves further research. Tremendous advances have been made in dissecting the transcriptional regulation mechanisms underlying stress responses. In contrast, related research on epigenetic modifications is still insufficient and lacks in-depth exploration. Accumulating evidence demonstrates that epigenetic modifications act as essential regulators when plants cope with diverse abiotic and biotic stresses. For this reason, it is imperative to systematically characterize the epigenetic regulatory patterns of core regulatory factors in future studies.

## 3. Retrograde Feedback Responses in the ER, Chloroplasts, and Mitochondria

In addition to their roles in cellular metabolism, the endoplasmic reticulum (ER), chloroplasts, and mitochondria function as signaling hubs that relay stress information to the nucleus to orchestrate the cellular heat stress response (Figure 2). Thus retrograde signaling pathways alleviate harmful effects within organelles.

### 3.1. Membrane-Associated Transcription Factors Relay Heat Signals via the Unfolded Protein Response

The ER, a central site for protein synthesis and folding, is particularly vulnerable under adverse stress. Adverse stress induces protein misfolding that triggers ER stress and activates the Unfolded Protein Response (UPR). Membrane-associated transcriptional factors play a pivotal role in this process by sensing ER stress and transducing signals to the nucleus, thus rearranging transcriptional reprogramming and relieving ER stress [23]. For instance, upon the accumulation of misfolded proteins, the ER membrane-localized transcription factor AtbZIP28 translocates to the Golgi apparatus. There, it is cleaved by site-1 and site-2 proteases (S1P and S2P), and then AtbZIP28 moves to the nucleus. Nuclear-localized AtbZIP28 recruits NF-Y subunits to form a functional complex, and upregulates ER stress-responsive genes [24,25,26,27].

A parallel UPR pathway involves Inositol Requiring Enzyme 1 (AtIRE1) and AtbZIP60. ER stress activates the ribonuclease AtIRE1, which catalyzes the unconventional splicing of AtbZIP60 mRNA, removing its transmembrane domain-encoding sequence. Thus, activated AtbZIP60 translocates to the nucleus and regulates ER stress-related genes independently or synergistically with AtbZIP28 [28,29].

Several UPR components are integral to thermotolerance. AtbZIP28 is essential for heat tolerance via upregulating heat-responsive genes like *HSP26.5* [25]. Its paralog, AtbZIP17, also contributes to maintaining fertility under thermal stress [30]. In rice, the transmembrane bZIP factor OsbZIP60 (encoded by OPAQUE3) operates through a conserved mechanism, activating genes for starch/protein synthesis and UPR targets to preserve ER homeostasis in endosperm cells during heat stress [31]. The UPR mediates vital retrograde signaling between the endoplasmic reticulum (ER) and the nucleus. This conserved protective pathway can be activated in response to a broad spectrum of adverse environmental stresses in plants. Despite the well-documented activation of UPR under various stressors, the functional conservation of UPR-associated genes across different stress contexts still requires further verification. Beyond the extensively characterized UPR, recent studies have identified independent unfolded protein response systems in chloroplasts (cpUPR) and mitochondria (UPRmt). These organelle-specific UPR pathways maintain internal protein homeostasis independently [32]. Currently, we know very little about how cpUPR and UPR^mt^ perceive heat stress signals and execute downstream responses. Further investigations are urgently needed to uncover their regulatory networks and biological significance under high-temperature conditions.

### 3.2. ROS Signaling Mediates Heat Responses in Chloroplasts and Mitochondria

Chloroplasts and mitochondria mainly function in photosynthesis and respiration separately. These processes trigger a surge in reactive oxygen species (ROS) and alter the redox state. Thus, chloroplasts and mitochondria are vital signaling organelles that emit retrograde signals to the nucleus.

Chloroplast-derived ROS, for instance, act as important retrograde messengers, activating nuclear genes encoding small heat shock proteins and cytosolic chaperones. In addition, ClpD, a chaperone subunit of the chloroplast Clp protease, is prone to precursor overaccumulation upon overproduction. Constitutive overexpression of ClpD induces chloroplast precursor overaccumulation (cPOS). This process modulates the heat shock response [33]. The protein GUN1 serves as a central integrator of multiple chloroplast retrograde pathways in Arabidopsis. It modulates chloroplast protein import by interacting with the chaperone cpHSC70-1 and influences nuclear photosynthetic gene expression [34]. GUN1 also binds plastid RNAs to promote their maturation, thereby coordinating chloroplast function with nuclear activity [35]. Recent research also verifies that GUN1 functions as a core component between chloroplast and nucleus communication via ROS signals in plant responses to heat. Loss of GUN1 leads to GOLDEN2-LIKE transcription factors 1/2 (GLK1/2); thereby, GLK1/2 promotes PhANGs (photosynthesis-associated nucleus-encoded genes) in response to heat-stressed plastids, leading to the accumulation of chloroplast-derived ROS. The nucleus-encoded chloroplast protein EX1 detects elevated levels of ^1^O_2_ and activates operational retrograde signaling to induce stress-related gene expression [36]. However, the mechanism by which EG1 maintains floral robustness is still unknown. In addition, chloroplast ribosomal protein S1 (RPS1) is a heat-responsive protein and functions in protein biosynthesis in the chloroplast. Disrupting RPS1 in Arabidopsis increases heat sensitivity and compromises the expression of the master regulator *HSFA2* [37]. The finding also establishes the linkage between chloroplast translation capacity and heat-responsive gene expression, indicating that RPS1 participates in retrograde signaling to sustain cellular homeostasis.

Mitochondria are also key sites for producing ROS. In rice, mitochondrial lipid metabolism contributes to thermotolerance. The mitochondria-localized lipase Extra Glume1 (EG1) helps maintain the normal expression pattern of key floral identity genes under heat stress, suggesting that mitochondria buffer reproductive development against temperature fluctuations [38]. ROS are pivotal signaling molecules that participate in retrograde signaling between chloroplasts, mitochondria and other cellular compartments. However, the generation, distribution and dynamic changes in ROS derived from chloroplasts and mitochondria are still not fully understood. Uncontrolled overaccumulation of ROS triggers oxidative stress and severely impairs normal cellular functions. Elucidating how plants precisely modulate ROS levels to strike a balance between signaling function and toxic effects will be a valuable direction for future research.

## 4. Future Perspectives

Although significant progress has been made in understanding organelle communication under heat stress, many important scientific questions remain unanswered. This article proposes that heat sensing primarily occurs at the plasma membrane (PM) and cytosol, after which heat signals are transmitted to the nucleus. However, all plant organelles are susceptible to heat stress. Certain proteins residing in these organelles may also act as potential thermosensors. Additional studies have demonstrated that the plant-specific mitochondrial fission machinery mediates responses to heat stress through piecemeal mitophagy in Arabidopsis [39]. This mechanism implies that mitochondrial proteins may act as potential heat sensors. Nevertheless, the way heat signals are perceived and transduced within mitochondria remains uncharacterized. In addition, the chloroplast-localized protein Multiple Organellar RNA-editing Factor 8 (MORF8) undergoes heat-dependent phase separation both in vivo and in vitro. This process inhibits RNA editing at multiple sites within the ndhD transcript, compromises NDH activity, and ultimately impairs photosynthetic efficiency [40]. Notably, it is still unclear whether other organelles possess independent thermosensing abilities. More importantly, it remains largely unclear how these putative organelle-localized sensor proteins transmit thermal signals across distinct organelles and coordinate to counteract heat-induced cellular damage. We observe that dynamic Ca^2+^ signals in the cytosol can transduce heat signals. However, other organelles including the ER also exhibit Ca^2+^ concentration waves under heat stress [41], yet their distinct heat-stress-responsive mechanisms remain largely uncharacterized. In addition, how Ca^2+^ signals coordinate responses across two or more organelles requires further investigation. Moreover, other chemical messengers such as cAMP also sense fluctuations across distinct organelles in the early stage of heat stress, thereby amplifying the initial signaling events. Notably, how alterations in these chemical molecules mediate inter-organellar communication remains unclear. These findings are important for avoiding heat stress in the early stage.

Plant heat responses range from thermoacclimation to programmed cell death: moderate heat activates adaptive protective pathways, and severe heat may eventually trigger ferroptosis [42]. Under mild heat conditions, multiple proteins undergo post-translational modifications or subcellular relocalization to initiate heat defense signaling—typical examples include DGK7 (subject to protein modification) as well as NTL3 (which shifts its subcellular localization upon heat exposure). These molecular events collectively enhance plant thermotolerance. Nevertheless, the regulatory mechanisms underlying plant responses to severe heat remain largely uncharacterized and require further investigation. Current research predominantly focuses on establishing regulatory networks between the nucleus and other organelles. Other organelles such as the ER, chloroplasts, and mitochondria have limited capacity to resolve heat stress autonomously; more research is needed to investigate the specific contributions of single organelles and to integrate multiple organelles into a global regulatory network. With the advent of single-cell sequencing and advanced in vivo imaging technologies, cell-type-specific organelle communication should be actively explored. In addition, the specific organelles at specific stages also deserve more research. For example, in developing grains, apart from most HSPs localizing in the ER, HSPs localized in other organelles such as the plastid are important for protein stability. For example, in rice, HS triggered the expression of *OsHSP60-3B* and *OsHSP40-1*, which are all plastid-localized HSPs that play a positive role in heat stress [43,44]. However, the upstream regulators of HSPs remain poorly understood. Beyond individual or pairs of organelles, the overall homeostasis of the organelle network is critical for plant thermotolerance. Membrane contact sites (MCSs) act as core platforms for inter-organelle communication, maintaining cellular homeostasis. Unlike animals, plants contain specialized organelles-chiefly plastids and a large central vacuole. In this organelle interactome, the endoplasmic reticulum (ER) serves as the central hub for lipid synthesis and trafficking, establishing functionally vital membrane contacts with numerous other organelles [45]. Collectively, these findings raise the hypothesis that MCSs could mediate signal exchange between plant organelles under stress conditions. Conventional approaches failed to achieve precise quantification and high-resolution visualization of inter-organellar contacts, which constitutes a major limitation of previous research. Thus, this field requires further in-depth investigation.

Above all, we summarize organelle communication mainly through heat sensing and signal transduction through the nucleus and other organelles. Moreover, we provide potential sensors as well as communication target proteins. Therefore, integrating genome editing, synthetic biology, and artificial intelligence (AI)-assisted design offers promising strategies to optimize these regulatory modules, thereby improving thermotolerance without incurring yield penalties.

## Data Availability

No new data were created or analyzed in this study. Data sharing is not applicable to this article.
